# An Effective and Safe Novel Treatment of Opioid Use Disorder: Unilateral Transcranial Photobiomodulation

**DOI:** 10.3389/fpsyt.2021.713686

**Published:** 2021-08-10

**Authors:** Fredric Schiffer, Alaptagin Khan, Elizabeth Bolger, Edward Flynn, William P. Seltzer, Martin H. Teicher

**Affiliations:** ^1^MindLight, LLC, Newton Highlands, MA, United States; ^2^Developmental Biopsychiatry Research Program, McLean Hospital, Belmont, MA, United States; ^3^Department of Psychiatry, Harvard Medical School, Boston, MA, United States

**Keywords:** opioid use disorder, opioid cravings, opioid use, hemispheric laterality, photobiomodulation

## Abstract

**Background:** The opioid epidemic is a global tragedy even with current treatments, and a novel, safe, and effective treatment would be welcomed. We report here our findings from our second randomized controlled trial to evaluate unilateral transcranial photobiomodulation as a treatment for opioid use disorder.

**Methods:** We enrolled 39 participants with active opioid cravings at 2 sites, 19 received the active treatment which consisted of a 4-min twice weekly (every 3 or 4 days) application of a light-emitting diode at 810 nm with an irradiance of 250 mW/cm^2^ and a fluence of 60 J/cm^2^ to the forehead over either the left or right dorsolateral prefrontal cortex with a fluence to the brain of 2.1 J/cm^2^. Twenty participants received a sham treatment with the same device with foil over the bulb. The side of the treatment was based on Dual-Brain Psychology, which posits that one hemisphere is more affected by past maltreatments and is more prone to anxiety and drug cravings that the other hemisphere. We treated the hemisphere with the more positive hemispheric emotional valence (HEV) by 2 tests for HEV.

**Results:** Our primary outcome was changes in pre-treatment opioid craving scale (OCS) minus baseline, and we found using a mixed model that the active group had a highly significant treatment ^*^ time benefit over the sham group, *p* < 0.0001, effect size at the last follow-up of 1.5. The active treatment benefited those not on buprenorphine as well as those not on it. The TimeLine Follow Back measure of opioid use was significantly better in the actively treated group, *p* = 0.0001, with an effect size of 0.45. We observed no adverse effects.

**Conclusion:** Active unilateral transcranial photobiomodulation to the brain hemisphere with the better HEV was better than sham in the reduction of opioid cravings and opioid use to a very significant degree in a RCT of 39 participants at 2 independent sites. In the active group those on buprenorphine and those not on it both had improvements in cravings over the study. No adverse responses were reported in either group. ClinicalTrials.gov Identifier: NCT04340622.

## Introduction

Opioid use disorder (OUD) is causing profound suffering, death, and destruction to individuals, families, and societies on a global scale. According to the National Institute on Drug Abuse (1), “The combined healthcare, crime-related, and productivity costs of tobacco, alcohol, and illicit drugs exceed $700 billion a year, but dollars only poorly approximate the devastating human cost of substance use disorders.” According to the CDC, opioids were involved in 42,249 deaths in 2016, a 28% increase over 2015 and 5-fold increase since 1999 (2). The mental suffering of drug abused patients and their families, the physical health complications, the loss of productivity, and the increase in criminality are all catastrophically injurious. Current treatments are obviously not stemming the tide of this disaster and there is a pressing need for additional stand-alone or add-on treatments that are safe and efficacious.

The current evidence-based standard for the treatment for OUD is medication management using buprenorphine (typically in combination with naloxone to prevent misuse) or methadone in relatively low doses to reduce opioid cravings and withdrawal symptoms. Buprenorphine and methadone are substantially more effective than placebo in retaining people in treatment and suppressing illicit opioid use (3, 4). However, there is considerable reluctance to use this approach in some quarters, often from an inaccurate concern that one is substituting one kind of addiction for another. Knudsen et al. (5) estimated that <50% of privately funded substance use disorder treatment programs offer medication management and only about a third of patients with opioid dependence at these programs actually receive it. Further, the dropout rate is high, with estimates that up to 40–50% of patients will discontinue medication management prematurely, frequently within the first month (6–8). Hence, there is a need for effective stand-alone treatments that would be acceptable to individuals and programs that eschew medication management and add-on treatments that can enhance the retention rates and abstinence rates of medication management.

Medication management makes sense pharmacologically but it does not address the underlying psychological and neurobiological factors that place individuals at risk. A complementary or alternative strategy emerges from the work of Schiffer on “Dual-Brain Psychology” (9–12). Based on his clinical experience and on the split-brain studies (13, 14), Schiffer has theorized that, in adulthood, maltreatments, and traumas, especially from childhood, become associated as a persistent trait with one brain hemisphere, either left or right, making the mind of that side immature and prone to seeing the world through the eyes of a traumatized child, affecting the person's affects, thoughts, and behaviors, generally in a negative manner. The other hemisphere has a mental perspective that is more mature and healthier. In his clinical practice, Schiffer uses lateral visual field stimulation to bring forth these different personalities. This is easily accomplished by restricting his patients' vision with taped goggles, their hands, or a letter size envelope to either the left hemifield of the left eye or the right hemifield of the right eye, which is accompanied by enhanced activation throughout the contralateral hemisphere (15). The method and its consequences are best described in his book, Of Two Minds (9), which using transcripts from patient sessions, demonstrated that looking out of one visual field vs. the other led to remarkable changes in personality, in many of his patients, such that he could have conversations with different personalities in the same person, depending on which visual field they were looking out of. Out of one visual field the patient might see Schiffer as harsh and critical as his father had been. He might tend to be critical of himself and if he had a history of drug abuse, he would likely develop drug craving looking out of that lateral visual field, while out of the opposite lateral visual field the patient would generally see Schiffer as supportive and see himself in a positive light. His drug craving would generally be greatly diminished or eliminated. Looking out the first visual field again would usually return the patient to his negative perceptions, thoughts, and actions. Schiffer could have different lengthy conversations with each side, and he would teach the patient to try to encourage the positive side to become more dominate and to recruit that side as a co-therapist in helping the more troubled side. Other than the fact that the lateral visual fields are neurologically connected to the contralateral hemisphere, he does not have a good explanation for his observations, why simple lateral visual stimulation can within seconds alter most patients' psychological state. This effect of lateral visual field stimulation was first reported by Wittling and Schweiger (16) and Wittling and Roschmann (17).

These observations were rigorously tested and well-supported with experiments at our laboratory at McLean Hospital with fMRI (15), near infrared spectroscopy (18), rapid Transcranial Magnetic (rTMS) electroencephalograms (19), evoked potentials (20), and psychometrics (11, 12, 19). Interestingly, while hemispheric valence theories delineate the right hemisphere as the more emotional and/or the more negative (21), Schiffer et al. (18) found that in about 45% of individuals that the more immature personality was actually associated with the left hemisphere. Schiffer proposed that difference in laterality would substantially affect the efficacy of lateralized treatments for depression with rTMS, and confirmed this in two studies (22, 23).

Schiffer then proposed that selectively stimulating the hemisphere associated with the more mature personality would be beneficial in alleviating symptoms of depression, anxiety or drug craving and explored this possibility using transcranial photobiomodulation (tPBM) (24).

Photobiomodulation, formerly called low-level light/laser therapy, is a burgeoning field, which has about 1,500 PubMed citations, most in recent years (25–27). Over decades, these therapies have been used mostly to treat wound healing, musculoskeletal disorder, and gastrointestinal disorders. In 2009 Schiffer and Hamblin and associates (24) performed the first use of transcranial photobiomodulation (tPBM) for the treatment of anxiety and depression. tPBM has been shown to activate mitochondria through near infrared absorption by cytochrome-C (28, 29), increase blood flow (24), integrate and segregate brain networks (30–32), and inhibit the default mode (33). tPBM (29, 34, 35) is a newer part of the burgeoning field of photobiomodulation. tPBM has been used for cognitive enhancement (36–38), sexual disorders (39), traumatic brain injury (31, 40, 41), depression and anxiety disorders (29, 42, 43) and is being explored for other neurological brain disorders (44–46). Zomorrodi and associates found that in volunteer participants tPBM at 810 nm induced significant EEG changes increasing the power of alpha, beta and gamma and decreasing lower frequencies in a blinded RCT. They also found evidence for improvements in neural connectivity. El Khoury reported (33) that tPBM, by MRI, inhibited the default network, and Figueiro Longo et al. (31) and associates treated patients with traumatic brain injury and found in serial DTI studies that the active group but not sham had marked improvements at 3-months in their fiber pathways, through re-myelinization. Recent work suggests that a membrane opsin, TRPV1 (47) and extracellular TGF-beta1 (48) appear to be involved in the anti-inflammatory effects of photobiomodulation.

We chose F3 or F4 in our initial study in 2009 (24) and a subsequent study (49) because we hypothesized that the dorsolateral prefrontal cortex in each hemisphere might activate a broader area of the irradiated hemisphere and evoke an experiential change. Our primary aim was to activate the hemisphere with the more positive HEV as we did with our lateral visual field test (9, 11, 12, 15, 18, 19, 22, 23). Our strong clinical results (24, 49, 50) suggest that we might have chosen well. We have not tested fp1 or fp2, which we believe might be inhibitory.

Schiffer decided to explore whether using tPBM on the forehead over the more mature hemisphere might lead to improvements in opioid use disorder patients in his private practice. His very positive findings (51) led him to lead the design and performance of an initial double-blinded randomized control trial (RCT) (49), which reported statistically significant positive results with an effect size of 0.73, showing decreased opioid cravings in a within subject design in which 17 participants received an active or a sham treatment at week 1 and the opposite treatment at week 2.

The aim of the present study was to assess in a two-center RCT whether there would be a greater reduction in opioid craving over 4-weeks of twice weekly treatments with active vs. sham tPBM, and to compare the degree of efficacy in participants receiving or not receiving medication management. The primary outcome measure selected *a priori* were ratings on the opioid craving scale and the expectation was that there would be a reduction in opioid cravings of at least 60% in the active group vs. about a 20% decrease in the sham treatment group. We decided to examine opioid use during the study, even though active use was not a requirement for enrollment.

## Materials and Methods

### Setting and Enrollment

This two site RCT took place at MindLight, LLC and in the Developmental Biopsychiatry Research Program at McLean Hospital, Harvard Medical School. The study was approved by the New England Regional IRB (now WCG IRB) and registered at clinicaltrials.gov, Identifier: NCT04340622. All participants provided informed written consent, The main inclusion criteria were a current or recent active opioid use disorder (OUD) diagnosis and current craving for opioids with ratings of at least 4 on the 10-point opioid craving scale (52) and age between 18 and 70 years. Exclusion criteria included past history of a psychotic disorder, violent behavior, past suicide gesture or attempt, current suicidal ideation, neurological disorders, pregnancy, or an inability to understand the consent process. Participants were screened, recruited and assessed independently at the two sites.

### Treatment

Each participant was given twice weekly 4-min treatments with either the active unilateral tPBM or its sham. For the active treatment we used a light-emitting diode (LED) at 810 nm with a HWFH of 40 nm (Marubeni America Corporation, 3945 Freedom Circle, Suite 1000, Santa Clara, CA 95054) which when applied to the skin had an irradiance of 250 mW/cm^2^. Our device is a prototype for which a Pre-submission has been submitted to the FDA. It can be replicated by an engineer using the Marubeni 810 nm LED and heat sink, a computer fan, and power supply. We are working with Vivonics, Inc., Bedford, MA to design and develop a commercially available device based on our prototype as discussed in the conflicts of interest section.

The treatment consisted of exposure to the light for 4-min at one of 2 sites on the forehead that correspond to the 10–20 EEG sites, F3, and F4, with a fluence of 60 J/cm^2^. Based on a penetration of 3.7% of the light to the dura (53), we applied 2.1 J/cm^2^ (with an irradiance of about 9 mW/cm^2^) to the treated area of the brain. Our level of light exposure is well below the ANSI RP-27 standard of 0.32 W/cm^2^. Barolet et al. (54) wrote, “Fluences in the range of tens of J/cm^2^ are likely to be protective and overall beneficial to the skin, while fluences in the range of hundreds of J/cm^2^ are likely to be damaging and overall deleterious to the skin. The same would apply for irradiance parameters.” The delivered fluence of our device to the skin is about 25% less than the device that was used in the study of 1,410 stroke patients without any observed side-effects. A safety study in rats showed that exposure to higher intensities (e.g., 10 X optimal) resulted in no discernible neurological deficits or evidence of histopathological damage at light or electron microscopy levels (55).

The sham treatment was identical to the active treatment except that the LED was covered with aluminum foil to prevent near infrared photons, but not warmth, from reaching the brain. The devices were cooled with a heat sink and a computer fan. The treating clinician applied the light to the participant in a manner that did not allow either the recording research assistant or the participant to see if the device was active or sham.

### Measures

The primary outcome measure was ratings of craving on the opioid craving scale (OCS) (52). The scale consists of three questions scored from 0 to 9 and averaged to provide a composite score. The Timeline Followback (56, 57) calendar method was used to determine the days, type, and amount of drug use during the preceding week. Drug use was also assessed by urine drug screen (CLIA Waived Inc. Instant Drug Test Cup II) at each visit. Ratings of depression and anxiety were assessed each week using the Hamilton Depression Rating Scale and the Hamilton Anxiety Scale (58, 59). An abbreviated Positive and Negative Affect Scale (PANAS) (60) and a Wellness and Distress 10-point scale, of our design, were also used to provide affect and well-being measures before and after each treatment. Two tests were used to determine which hemisphere had the more positive affect. The first was a lateral visual field test in which participants partially blocked their vision so that they could see out of only their right lateral or left lateral hemi visual field at a time while viewing photographs of an angry man and rating their levels of anxiety and opioid craving. This test takes 1 min. The second test was a 2.5 min computer test for hemispheric emotional valence (CTHEV), which presented images of angry men to one visual field and then the other while participants rated their emotional response. The hemisphere contralateral to the visual field with the lowest ratings of anxiety and cravings was designated as the more positive hemisphere, and this is the hemisphere to which the active or sham tPBM was applied.

### Sequence of Events

Participants were phone screened for eligibility. Those who appeared eligible were invited to the laboratory, had the study fully described, signed an informed consent agreement, participated in an assessment of their psychiatric, medical and drug use history and were then randomize to active or sham treatment groups.

Each participant then moved into the 4-week treatment phase in which they received two treatments each week spaced 3 or 4 days apart. The visit began with a urine drug screen for all participants and a pregnancy test for females. This was followed by pre-treatment assessments consisting of drug use determination via Timeline Followback, Hamilton Depression and Anxiety ratings, the OCS, PANAS, Wellbeing/Distress Scale, the Lateral Visual Field Test and the CTHEV. Participants then received 4-min of active or sham tPBM directed into the more positive hemisphere at frontal positions F3 or F4. This was then followed by an immediate post-treatment evaluation phase during which time the OCS, PANAS, Wellbeing/Distress Scale, Lateral Visual Field Test and the CTHEV were repeated.

Three post-treatment visits spaced 1 week apart then followed the treatment phase. Each visit consisted of a urine drug screen, Timeline Followback, Hamilton Depression and Anxiety ratings, OCS, PANAS, Wellbeing/Distress Scale, Lateral Visual Field Test and CTHEV.

### Statistical Approaches

The statistical analyses were conducted using JMP 15.2.1 (61) or R 4.0.3 (62). Our primary measure of craving was the OCS score minus the baseline score. This was chosen because the raw OCS scores were not normally distributed and when transformed by subtracting the baseline, the distributions became normal. Secondly, we felt that the OCS minus the baseline score directly measured the change in OCS that was due to the treatments. For these measures we used mixed models with OCS-baseline as the dependent variable and for the repeated measures correlations we used either a first order autoregressive (AR1) or first or second order autoregressive moving average (ARMA1, ARMA2) covariance structures. We included random intercepts and slope for treatment, site and participants. For fixed effects we first entered a full factorial model that included treatment, time, study site and buprenorphine. We also included covariates for gender, ethnicity, employment, ACE trauma score and handedness following the European guideline on adjustment for baseline covariates in clinical trials (63). We selected the best-fitting parsimonious model by sequentially removing factors that had a *p* > 0.05 and whose removal did not significantly worsen overall fit. From the final model we had a least square mean for Treatment (active vs. sham) and for Treatment ^*^ Time. We found that for all of our other outcome measures the raw data were not normally distributed, but the transformed data, by subtracting the baseline, was, and so in all of our mixed model analyses we used the same procedures as with our OCS analyses.

## Results

### Participants

All participants who passed the phone screen and came in for the initial interview were accepted and gave written informed consent. Most participants at MindLight were recruited from an advertisement on Craigslist, but 4 came from referrals from drug clinics. At McLean, 5 participants came from Craigslist, 4 from drug clinic referrals, 3 from Partners Rally recruitment site, and 3 through friends of participants. The participants' demographic information is presented in Table 1.

**Table 1:** Demographic information for participants.

		***N***	**Gender**	**Ethnicity**	**Age**	**Handedness**	**Grade**	**ACE**	**Employment**	**Buprenorphine**
**MindLight**										
	Active	13	2 f, 11 m	5 b, 8 w	44 ± 8.7	4 l, 9 r	12 ± 3.0	4.8 ± 2.6	3 y, 10 n	7 n, 6 y
	Sham	11	1 f, 10 m	6 b, 5 w	46 ± 12.0	2 l, 9 r	18 ± 15.1	4.7 ± 3.2	4 y, 7 n	10 n, 1 y
**McLean**										
	Active	7	1 f, 6 m	3 b, 4 w	47 ± 13.8	1 l, 6 r	14 ± 1.9	4.0 ± 2.0	1 y, 6 n	5 n, 2 y
	Sham	8	4 f, 4 m	2 b, 6 w	44 ± 14.8	1 l, 7 r	13 ± 2.1	4.8 ± 2.3	4 y, 4 n	3 n, 5 y

*The groups were formed by randomization at the 2 sites and are not exactly matched, but by mixed model analysis none of these demographic parameters were significant enough to be included as predictors in the model. Age, Grade, and ACE scores are mean ± SD. f, female; m, male; b, black; w, white*.

The groups were formed on a first come basis by randomization (by random numbers) at the 2 sites and are not exactly matched, but by mixed model analysis none of these demographic parameters were significant enough to be included as predictors in the model.

### Cravings

Our primary outcome was the response of participants to the Active and Sham treatments as measured by the OCS, a 10-point, 0–9 scale.

**Figure 1:**
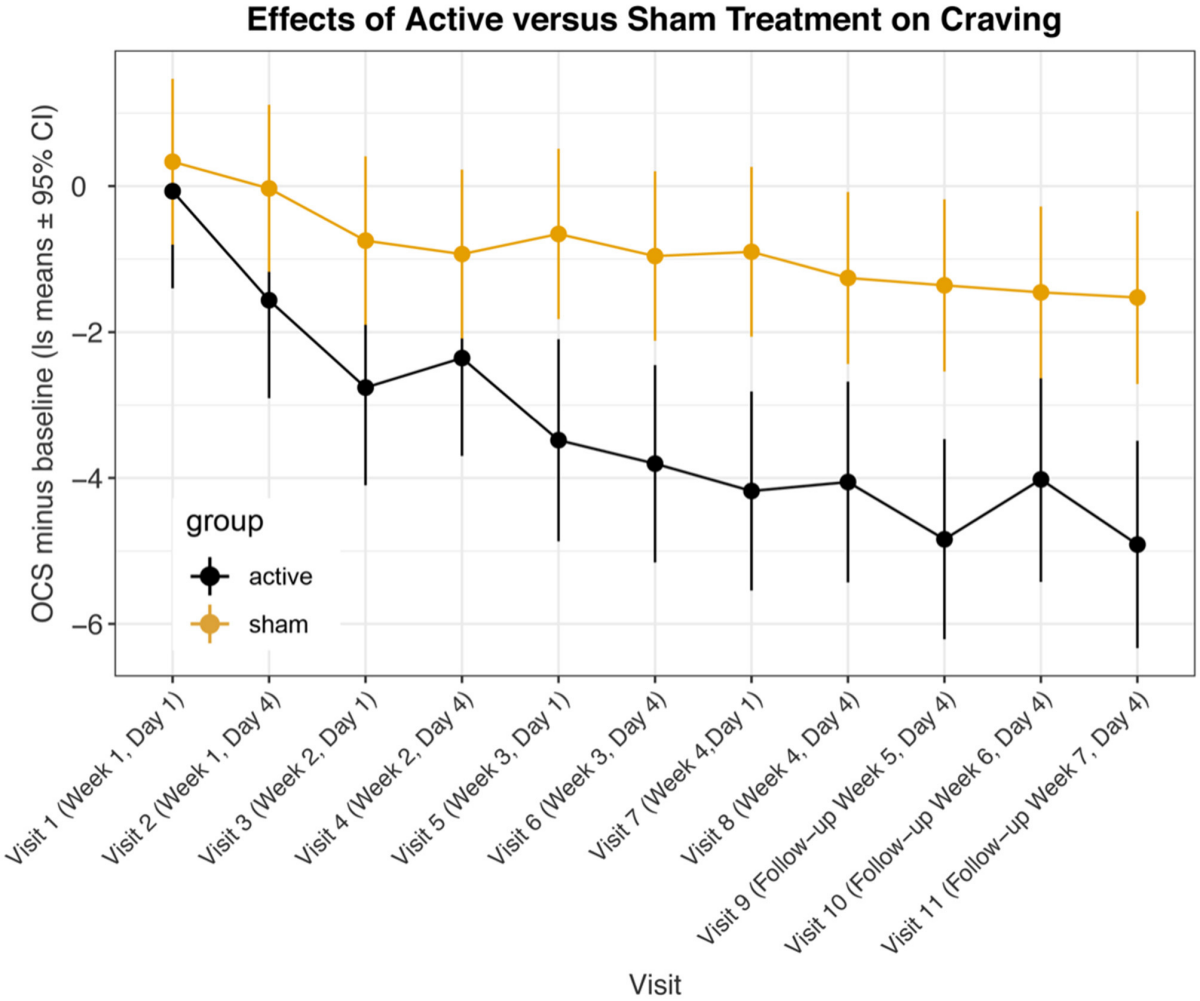
Least square means for opioid craving scores minus baseline from mixed model comparing active vs. sham treatments across visits. Data are means ± 95% confidence intervals.

**Figure 2:**
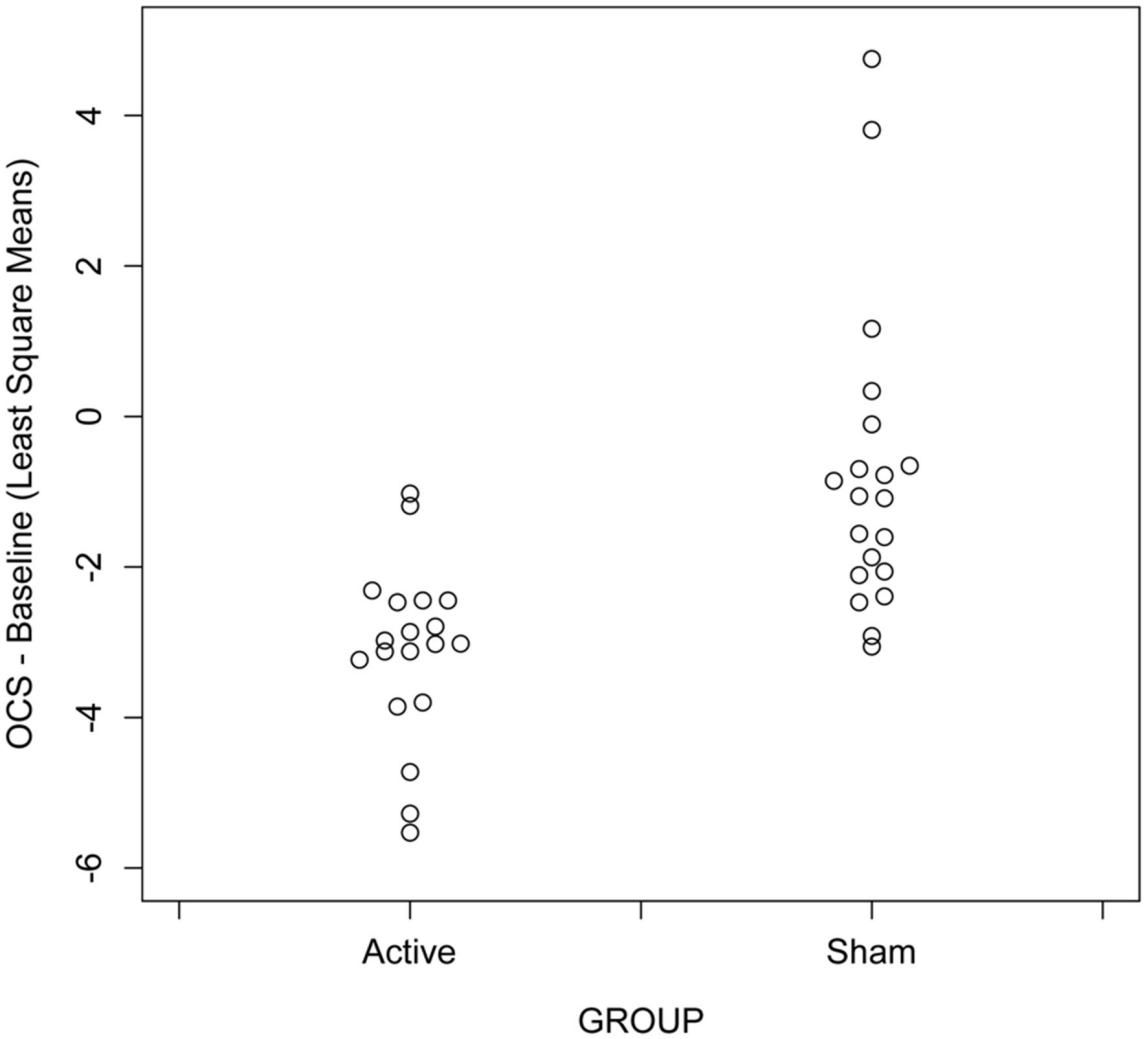
Scatter plots showing differences across visit in opioid craving scores in participants receiving active vs. sham treatment with unilateral transcranial photobiomodulation from mixed model analysis, *N* = 39 with 20 actives.

We recorded the OCS both before and after each treatment visit. We felt that the initial OCS scores before each treatment offered the best indication of the lasting effects of the treatment and we used this as our primary outcome. Figure 1 and Table 2 show the mixed model results for the initial OCS score minus baseline for “treatment ^*^ time,” which show that from visit 5 through visit 11 there were highly significant differences with large effect sizes for each of these visits between the active and sham groups. Figure 2 shows that by a mixed model analysis the overall treatment effect on initial OCS score was significantly better for the active vs. sham treatment, *p* = 0.0004, effect sized = 0.77, 19 active and 20 sham. Table 3 shows the percent improvement in cravings by a mixed model analysis. The active group had a 71% improvement in cravings form the 1st visit to the 3rd follow-up at visit 11, and the sham group had a 35% improvement. Overall, there was a 35% greater decrease in OCS scores in participants receiving active vs. sham treatment (*p* = 0.005).

**Table 2:** Least square mean differences between participants receiving active vs. sham treatment across visits by mixed model.

**Visit**	**Contrast**	**Difference**	**Std. error**	***t* ratio**	***p*-value**	**Lower 95%**	**Upper 95%**	**Feingold d**
1	Active-sham	−0.404	0.863	−0.467	0.6406	−0.4	0.86	−0.18
2	Active-sham	−1.532	0.87	−1.76	0.0793	−1.53	0.87	−0.67
3	Active-sham	−2.012	0.873	−2.305	0.0218	−2.01	0.87	−0.88
4	Active-sham	−1.424	0.875	−1.627	0.1048	−1.42	0.88	−0.62
5	Active-sham	−2.827	0.893	−3.164	0.0017	−2.83	0.89	−1.24
6	Active-sham	−2.845	0.88	−3.234	0.0013	−2.84	0.88	−1.24
7	Active-sham	−3.278	0.885	−3.703	0.0003	−3.28	0.89	−1.43
8	Active-sham	−2.797	0.895	−3.124	0.0019	−2.8	0.90	−1.22
9	Active-sham	−3.48	0.893	−3.896	0.0001	−3.48	0.89	−1.52
10	Active-sham	−2.563	0.905	−2.831	0.0049	−2.56	0.91	−1.12
11	Active-sham	−3.384	0.913	−3.709	0.0002	−3.38	0.91	−1.48

**Table 3:** Percent decrease in opioid craving score from visit 1 to 11 by mixed model analysis.

**Treatment**	**Estimate**	**Std. error**	**DF**	**Lower 95%**	**Upper 95%**	
**LEAST SQUARE MEANS ESTIMATES**						
Active	71.30%	10.19	156	50.69%	92.0%	
Sham	35.39%	8.18	160	18.26%	52.5%	
**Treatment**	**Difference**	**Std. error**	***t*** **ratio**	**Prob>|t|**	**Lower 95%**	**Upper 95%**
**STUDENT's t PAIRWISE DIFFERENCES**						
Active-sham	35.02%	12.9	2.72	0.005	9.71%	60.3%

**Figure 3:**
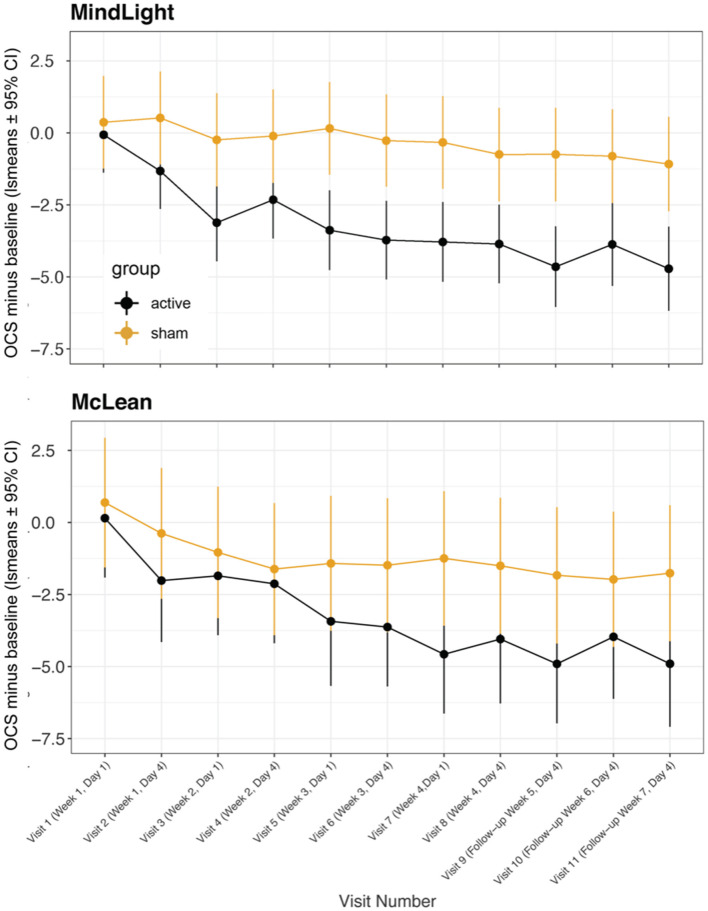
Least square means for opioid craving scores minus baseline from mixed model comparing active vs. sham treatments across visits at MindLight site vs. McLean site. Data are means ± 95% confidence intervals. Overall, there were no significant differences between sites or significant site by visit or treatment interactions.

**Figure 4:**
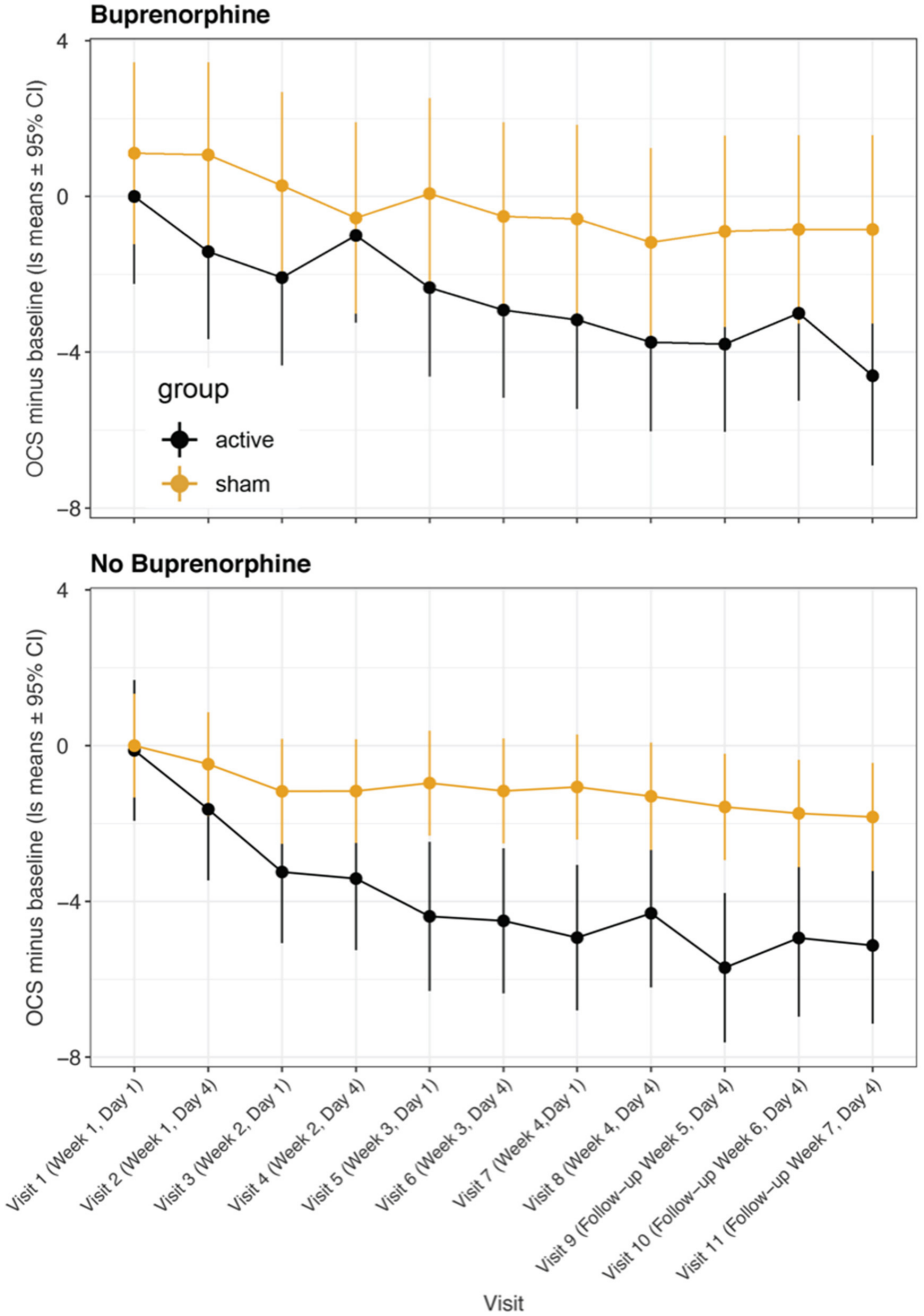
Least square means for opioid craving scores minus baseline from mixed model comparing participants receiving buprenorphine/suboxone vs. participants not receiving medication management. Data are means ± 95% confidence intervals. A significant treatment:visit interaction was present in both groups of participants.

In a previous randomized controlled study (49), we found that greater improvement occurred a week after treatment than immediately after, and in the present Phase I study, we found that the improvements 3 or 4 days after treatment were greater than those immediately after treatment. Figure 3 shows a comparison of OCS—baseline ratings at MindLight *N* = 24 and McLean *N* = 15, by mixed model analysis. Including treatment site as a main and interactive effect did not improve the fit (LR Test = 13.10, 0.931) and differences between sites could have easily occurred by chance. Figure 4 shows the results of a mixed model comparing Active v. Sham, on and off buprenorphine. As expected, in participants off buprenorphine there was a significant Treatment x Visit interaction [*F*_(11, 197_ = 2.14, p = 0.019] in the mixed model. This was also true for participants on buprenorphine [*F*_(11, 102)_ = 1.99, *p* = 0.037], even though only 14 participants were receiving buprenorphine, 8 Active and 6 Sham. Among the 15 active participants who completed the 3rd follow-up, the percent improvement from baseline for those (*N* = 7) on buprenorphine was 63% ± SD 0.24, and for those (*N* = 8) not on buprenorphine it was 79% ± SD 0.21, *p* = 0.0001 by 2-sided Wilcoxon test.

The full mixed models and data set are presented in the Supplementary Materials.

### Opioid Used During the Study

**Figure 5:**
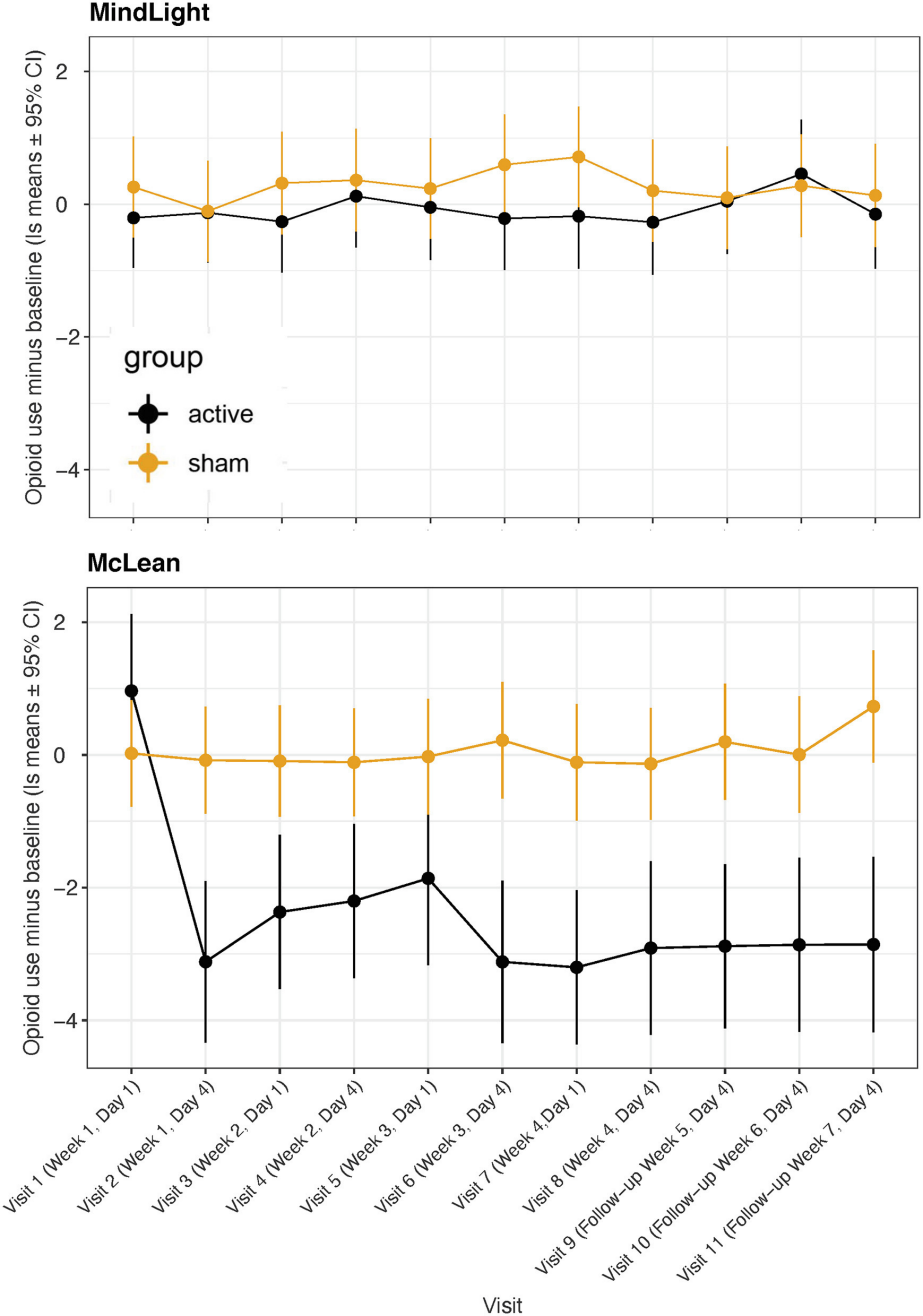
Least square means for Opioid Use (days * amount) minus baseline from TimeLine FollowBack across visits for active vs. sham treatment at MindLight and McLean sites.

Our primary measure of opioid use was the TimeLine FollowBack calendar method in which patients recalled their days and amounts of use. There were significant effects of treatment:visit [*F*_(10, 292)_ = 7.15, *p* < 0.0001], visit:site [*F*_(10, 292)_ = 7.38, *p* < 0.0001] and treatment:visit:site [*F*_(10, 292)_ = 5.28, *p* < 0.0001]. As seen in Figure 5 there was a substantial reduction in degree of use at the McLean site but not at MindLight. This was likely a consequence of a greater percent of participants using at baseline at McLean vs. MindLight (33 vs. 21%). Overall, there was significantly less use in the active group even though only 10 participants used opioids in the days prior to treatment, *p* < 0.0001. Feingold's d, effect size = 0.45. The second measure of Opioid Use was the days of use minus the baseline and in the active group there was an improvement from baseline of −81 days but a worsening in the sham group of +8. The statistical analysis of this parameter by a 2-sided Wilcoxon Rank Sum Test had a *p* = 0.009. The third measure of Opioid Use is the number of positive twice weekly urine screens. The active group had 8 that were positive, and the sham had 20, which by a 2-sided Wilcoxon Sign Rank Test for the positive urine screens had a *p* = 0.025.

The fourth measure of Opioid Use is retention. In both the active and sham groups at both sites the retention was very high. In the active group one patient dropped out because of a parole violation unrelated to the study. Another active participant completed 7 visits and was 2.33 at baseline and 7 at visit 2, then went to 0 cravings for 5 visits. He reported that he dropped out because of “family problems.” One sham patient came for one visit and did not return. The majority of the participants enjoyed coming to the study and most of the participants in the sham group reported at the end of the study that they thought they had received the active treatment.

### Symptom Ratings

**Figure 6:**
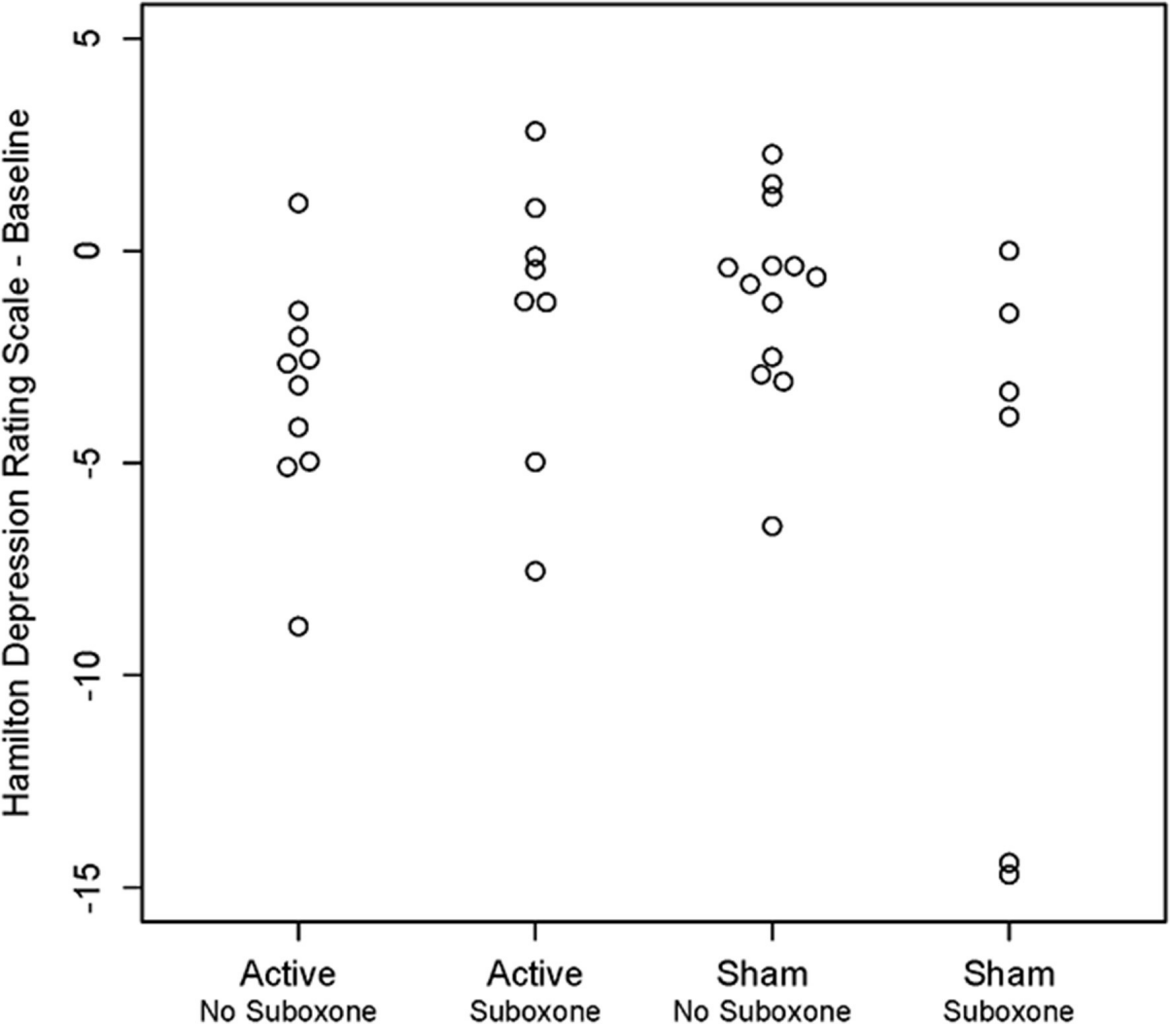
Scatter plot showing individual results for participants on and not on medication management with buprenorphine/suboxone following active vs. sham treatment with unilateral transcranial photobiomodulation. Note unexpected reduction in Hamilton Depression Rating Scale scores in two participants on buprenorphine who received sham treatment.

Mixed model analysis of the HDRS indicated that there was a significant triple interaction between visit:treatment:suboxone [*F*_(22, 292)_ = 1.59, *p* = 0.049]. As seen in Figure 6, two of the six sham participants on buprenorphine had extremely positive HDRS improvements. Among those not on buprenorphine there was a significant treatment:visit interaction [*F*_(11, 194_ = 2.06, *p* = 0.025]. Mixed model analysis of the HARS indicated that there were no significant main or interactive effects of Site or suboxone and the visit:treatment interaction fell short of significance [*F*_(11, 314)_ = 1.69, *p* = 0.075].

### PANAS Scores

Ratings of positive affect on the PANAS minus baseline did not vary by visit and there was no significant main effect of treatment [*F*_(1, 35)_ = 2.11, *p* = 0.16]. Similarly, ratings of negative affect on the PANAS did not vary by visit or treatment. The significant predictors were baseline score [*F*_(1, 332)_ = 44.10, *p* < 0.0001] and ethnicity [*F*_(1, 35)_ = 6.17, *p* = 0.018].

### Wellness and Distress Scales

Ratings of Wellness minus baseline did not vary by visit or site and there was no main or interactive effect of treatment (LR test = 0.57, *p* = 0.45). On the other hand, there was a significant visit:treatment interaction on ratings of Distress minus baseline [*F*_(11, 332)_ = 2.61, *p* = 0.0034] in favor of the active treatment.

## Discussion

The results reported here offer evidence that unilateral tPBM reduces opioid cravings and use and appears effective in our study in participants both on and off buprenorphine. These finding are consistent with our earlier RCT of unilateral tPBM for opioid cravings (49) and with its off-label use in a clinical practice (51) of OUD. We observed no side-effects, and none were reported as is usual in the literature (28, 29, 64), with one exception, in which a statistically significant, but not clinically significant, 6-point ± SD = 7 increase in diastolic blood pressure was observed over the course of an 8-week tPBM study with an N of 9 in the active group (65), compared with a decrease of 6 points ± 7 in their sham group *N* = 9.

The primary milestone was that active treatment would be associated with a 60% decrease in OCS ratings vs. a 20% decrease in the sham group. Mixed model test results (active = 71.3% ± SE 10.2% vs. sham 35.4 ± 8.2%) exceed the milestones by 11% for the active group and underestimated the response for the sham group by 15 percentage points. Still the difference in improvements in cravings for the active group over sham was highly significant and had a high effect size for treatment groups by visits. The overall difference between the active and sham groups was also highly significant.

Fudala and associates (66) reported a 4-week treatment study of buprenorphine vs. placebo and from their graph it appears that there was a 40% decrease in cravings at week 4 in the active group. In the present study we found a 64% ± SD 26% decrease in cravings in our active group, pre-treatment at the second session in week 4. Their study involved 326 participants and they did not provide exact numbers nor standard deviations, so a statistical comparison between the two studies is not possible, but our results seem at least comparable to theirs. Orman et al. (67) and Hew et al. (68) in a more recent reviews of buprenorphine cite Fudala's study.

In our Aims we did not consider the impact of buprenorphine but *post-hoc* we brought up the question of whether tPBM was a stand-alone treatment or an add-on, and whether tPBM was needed because we already have a treatment for OUD, buprenorphine. Although our inclusion criteria required that the participant have 4/10 craving to be enrolled, participants were allowed to be in treatment with buprenorphine, but not required to. We found that in the active group that there was a steady decrease in cravings over the treatment period in both participants not receivieng buprenorphine (*N* = 11) and these in treatment on buprenorphine (*N* = 8), which did not differ statistically. That is our results showed that participants treated with unilateral tPBM who were on buprenorphine had a steady further reduction in cravings, down an additional 65% over the course of the study and the treatment:visit interaction was significant. Thus, unilateral tPBM appeared to reduced cravings both in those on and those not on buprenorphine. None of these patients received any psychotherapy or other intervention during the study. In private practice, Schiffer (51), reported that combining unilateral tPBM with buprenorphine and integrated with his in-depth psychotherapy was extremely effective in treating patients with OUD.

Unilateral tPBM has advantages over buprenorphine. It is a non-drug treatment and therefore has no drug side-effects or interactions, no drug withdraws, no precipitated opioid withdraws, and no diversion risk. As a non-drug treatment, it may have broader acceptance.

Our second milestone was that active tPBM would reduce opioid use by 40% more than sham treatment. Least square means for days of use were reduced from baseline by 34 and 40%. Eighty-two and ninety-two percent on visits 8–11 in the active treatment group vs. 19, 14, 13, and 1% in the sham treated group. Those in the active group not on suboxone had a significant decrease on this measure of use (*N* = 11, *p* = 0.008), but there was no improvement among those (*N* = 8, *p* = 0.96) on buprenorphine. This was probably a floor effect as only two of the eight participants on suboxone were using opioids at baseline. However, Marcovitz and associates (69) reported that about 50% of patients on buprenorphine relapse over 1 year, and it seems that buprenorphine works well when patients are taking it, but 50% drop out and likely use. Ninety percent relapse after a medical taper (69).

Our other measures for use, days of use and positive urine screens, also showed a large advantage for the actively treated unilateral tPBM patients over sham.

### Psychiatric Rating Scales

Among the psychiatric rating scales there was a significant reduction in HDRS for participants not on buprenorphine and there was a treatment:visit interaction on the HARS that fell short of significance. In 2 earlier clinical trial participants showed strong positive responses on the HDRS and HARS (24, 49). There were no significant treatment:visit effects on ratings of positive or negative affect or wellness, but for distress there was a significant treatment:visit effect favoring the active treatment.

### Dual-Brain Psychology

Dual-brain psychology was described briefly earlier. This theory was the basis for our using unilateral tPBM to the hemisphere with the more positive HEV, and although this work has not been widely appreciated by clinicians and the academy, this report and the many others cited earlier strongly support its premises. The theory is so different from prior neuroscience and psychological theories that focused on small integrated brain areas relating to psychological function, and more similar to current models that focus on extensive networks. However, it goes much further by suggesting that the entire hemisphere is associated with mental properties, that is a mind or a personality, that is more affected by past maltreatments and traumas while the other hemisphere becomes associated with a healthier personality. The idea that we can have two minds each associated with one hemisphere, left or right (and not having all negative attributes associated with the right hemisphere) is not within our personal experiences, but requires an in-depth psychotherapy aided by hemispheric stimulation to be made apparent. Taking this view seriously requires reassessing our entire thinking about the brain and its relation to psychological states. This study and those that preceded it including, an fMRI study (15) showing that looking out each visual field robustly activates the contralateral hemisphere are strongly supportive of the hypothesis. Another 2 studies showed that the side on which a person feels more depressed by looking out of one visual field and then another, robustly predicted subsequent outcomes to a 2-week course of rTMS at 2 sites (22, 23). A report from a private practice (51) showed that unilateral tPBM was superior to bilateral tPBM, and a sub-study within an RCT (49) showed that cravings were significantly reduced more immediately after active treatment to the positive hemisphere than the negative, but no difference was found after sham treatments. So, we feel that Dual-brain psychology was not only the basis for the conception and design of this study, but it was also affirmed by the study.

Why or how photobiomodulation or lateral visual field stimulation induces in many patients a distinct change in personality is not yet understood, except that in both treatments we seem to be stimulating one brain hemisphere (15, 19, 22, 23) and with that inducing subjective experiences that are associated with it. We consider this an important discovery which we hope will lead to studies into the processes involved. Schiffer has speculated that tPBM might affect the brain biophoton information and thereby impact subjective experiences (70, 71), but this hypothesis is just speculative and will require creative testing.

## Conclusion

Active unilateral transcranial photobiomodulation to the brain hemisphere with the better HEV was superior to sham for the reduction of opioid cravings and opioid use to a highly significant degree in a RCT of 39 participants at 2 independent sites. In the active group those on buprenorphine and those not on it both had improvements in cravings over the course of the study. No adverse responses were reported in either treatment group.

## Data Availability Statement

The original contributions presented in the study are included in the article/Supplementary Material, further inquiries can be directed to the corresponding author/s.

## Ethics Statement

The studies involving human participants were reviewed and approved by WCG IRB and Partners/McLean IRB (ceded). The patients/participants provided their written informed consent to participate in this study.

## Author Contributions

FS and MT contributed to the design of the study. FS, MT, and WS contributed to the writing of the manuscript. EF, EB, and AK contributed to the data acquisition. All authors contributed to the article and approved the submitted version.

## Conflict of Interest

FS is the Founder of MindLight, LLC, which intends further research and commercialization of the methods and device described in the paper. The author has been issued 2 US patents which cover the method of unilateral tPBM to a positive hemisphere as described in this study: U.S. Patent No. 8303636, Methods for treating psychiatric disorders using light energy. Issued 11/06/2012, and U.S. Patent No. 8574279, Methods for treating psychiatric disorders using light energy. Issued 11/05/2013. He has filed on December 5, 2019 a US patent application, #16/703,937, Method and Apparatus for Determining Hemispheric Emotional Valence, and on August 3, 2020, he filed a US provisional patent application #63060177, Enhanced Treatment of Brain Disorders Utilizing Coordinated Negative Suppressive Stimulation and Related Devices Designed to Achieve Treatment. EF and WS were employed by MindLight, LLC. The remaining authors declare that the research was conducted in the absence of any commercial or financial relationships that could be construed as a potential conflict of interest.

## Publisher's Note

All claims expressed in this article are solely those of the authors and do not necessarily represent those of their affiliated organizations, or those of the publisher, the editors and the reviewers. Any product that may be evaluated in this article, or claim that may be made by its manufacturer, is not guaranteed or endorsed by the publisher.
