# Supplementary material for: An Effective and Safe Novel Treatment of Opioid Use Disorder: Unilateral Transcranial Photobiomodulation
Source: Front Psychiatry. 2021 Aug 10;12:713686. doi: 10.3389/fpsyt.2021.713686 (PMC8382852; doi:10.3389/fpsyt.2021.713686)
Supplement: Supplementary file 2 [file Table_2.DOCX]

**Supplement**

**An effective and safe novel treatment of opioid use disorder: Unilateral transcranial photobiomodulation**

Fredric Schiffer, MD^1,2,3^

Alaptagin Khan, MD^2,3^

Elizabeth Bolger^2^

Edward Flynn^1^

William P. Seltzer^1^

Martin H. Teicher, MD, PhD^2,^

**Linear Mixed Effects Models**

**1. Opioid Craving Scale**

This is the best fitting most parsimonious models. Factors that were eliminated included: Age, Gender, Ethnicity, Site, baseline craving, suboxone and main effect of treatment. This model used a second or autoregressive moving average covariance structure.

Variables

+ OCS.Pre.Base - opioid craving score, first rating for each visit minus baseline rating. (numeric)

+ Pt_ID - subject ID number (factor)

+ visitid_distinct - visits not including baseline, visit 1-8 Rx, 9-11 followup (integer)

+ visit – created as a factor (factor)

+ Treatment – active vs sham (factor)

+ suboxone - yes or no, factor

+ OCS.baseline - baseline OCS obtained prior to initiation of treatment (numeric)

+ Site – MindLight or McLean (factor)

+ Age - numeric

+ Gender – m or f (factor)

+ Ethnicity – w or b (factor)

+ Employment – n or y (factor)

+ Education.grade – range 4 – 18 (integer)

weighted <- lme(OCS.Pre.Base ~ visit + visit:Treatment, random = ~ 1|Pt_ID, correlation=corARMA(form = ~visitid_distinct|Pt_ID,p=2), weights = varIdent(form = ~1 | Treatment), method = "ML", data= f1, na.action = na.exclude)

numDF denDF F-value p-value

(Intercept) 1 320 0.012109 0.9124

visit 10 320 6.977082 <.0001

visit:Treatment 11 320 3.110544 0.0005

summary(weighted)

Linear mixed-effects model fit by maximum likelihood

Data: f1

AIC BIC logLik

1475.317 1581.701 -710.6583

Random effects:

Formula: ~1 | Pt_ID

(Intercept) Residual

StdDev: 1.791158 2.125793

Correlation Structure: ARMA(2,0)

Formula: ~visitid_distinct | Pt_ID

Parameter estimate(s):

Phi1 Phi2

0.5426455 0.1466283

Variance function:

Structure: Different standard deviations per stratum

Formula: ~1 | Treatment

Parameter estimates:

active sham

1.0000000 0.7735367

Fixed effects: OCS.Pre.Base ~ visit + visit:Treatment

Value Std.Error DF t-value p-value

(Intercept) -0.070175 0.6570310 320 -0.106807 0.9150

visit2 -1.493541 0.4361276 320 -3.424550 0.0007

visit3 -2.688695 0.5127319 320 -5.243861 0.0000

visit4 -2.284433 0.5760094 320 -3.965965 0.0001

visit5 -3.412080 0.6366298 320 -5.359598 0.0000

visit6 -3.733617 0.6473450 320 -5.767585 0.0000

visit7 -4.107824 0.6733688 320 -6.100408 0.0000

visit8 -3.985241 0.6939194 320 -5.743089 0.0000

visit9 -4.769072 0.7008377 320 -6.804817 0.0000

visit10 -3.949129 0.7247616 320 -5.448866 0.0000

visit11 -4.840713 0.7365890 320 -6.571796 0.0000

visit1:Treatmentsham 0.403509 0.8634045 320 0.467346 0.6406

visit2:Treatmentsham 1.531550 0.8699823 320 1.760438 0.0793

visit3:Treatmentsham 2.012126 0.8730552 320 2.304695 0.0218

visit4:Treatmentsham 1.423708 0.8752831 320 1.626568 0.1048

visit5:Treatmentsham 2.826856 0.8933255 320 3.164419 0.0017

visit6:Treatmentsham 2.845459 0.8798187 320 3.234142 0.0013

visit7:Treatmentsham 3.278225 0.8852297 320 3.703248 0.0003

visit8:Treatmentsham 2.796635 0.8951504 320 3.124207 0.0019

visit9:Treatmentsham 3.479540 0.8929964 320 3.896478 0.0001

visit10:Treatmentsham 2.563367 0.9054710 320 2.830977 0.0049

visit11:Treatmentsham 3.384436 0.9125784 320 3.708652 0.0002

**2. Opioid Craving Scale with Site**

This model is the best fitting model that includes treatment site. However, this model is not significantly better than the more parsimonious model that excludes site. This model used a second or autoregressive moving average covariance structure.

ocs.site.ARM2.weighted <- lme(OCS.Pre.Base ~ visit + visit:Treatment + visit:Treatment:Site, random = ~ 1|Pt_ID, correlation=corARMA(form = ~visitid_distinct|Pt_ID,p=2), weights = varIdent(form = ~1 | Treatment), method = "ML", data= f1, na.action = na.exclude)

numDF denDF F-value p-value

(Intercept) 1 299 0.0941227 0.7592

Visit 10 299 2.5901738 0.0050

Site 1 36 0.0369518 0.8486

suboxone 1 36 2.1309859 0.1530

Visit:Site 10 299 0.6384284 0.7805

Visit:Site:Treatment 22 299 2.0346570 0.0046

summary(ocs.site.ARM2.weighted)

Linear mixed-effects model fit by maximum likelihood

Data: f1

AIC BIC logLik

1506.221 1699.289 -704.1103

Random effects:

Formula: ~1 | Pt_ID

(Intercept) Residual

StdDev: 1.805309 2.089107

Correlation Structure: ARMA(2,0)

Formula: ~visitid_distinct | Pt_ID

Parameter estimate(s):

Phi1 Phi2

0.5533004 0.1313528

Variance function:

Structure: Different standard deviations per stratum

Formula: ~1 | Treatment

Parameter estimates:

active sham

1.0000000 0.7686479

Fixed effects: OCS.Pre.Base ~ visit + visit:Treatment + visit:Treatment:Site

Value Std.Error DF t-value p-value

(Intercept) 0.000000 1.1987371 298 0.000000 1.0000

visit2 -2.162770 0.8193501 298 -2.639617 0.0087

visit3 -2.000000 0.9215872 298 -2.170169 0.0308

visit4 -2.277778 1.0330664 298 -2.204871 0.0282

visit5 -3.562690 1.1844792 298 -3.007811 0.0029

visit6 -3.777778 1.1552251 298 -3.270166 0.0012

visit7 -4.722222 1.1906349 298 -3.966138 0.0001

visit8 -4.190192 1.2887575 298 -3.251342 0.0013

visit9 -5.055556 1.2341399 298 -4.096420 0.0001

visit10 -4.179353 1.2870103 298 -3.247335 0.0013

visit11 -5.151344 1.3098948 298 -3.932639 0.0001

visit1:Treatmentsham 0.740741 1.4732764 298 0.502785 0.6155

visit2:Treatmentsham 1.835025 1.5071935 298 1.217511 0.2244

visit3:Treatmentsham 1.011853 1.4857969 298 0.681017 0.4964

visit4:Treatmentsham 0.711714 1.4884760 298 0.478150 0.6329

visit5:Treatmentsham 2.190849 1.5675822 298 1.397598 0.1633

visit6:Treatmentsham 2.354344 1.5012425 298 1.568263 0.1179

visit7:Treatmentsham 3.536474 1.5034882 298 2.352180 0.0193

visit8:Treatmentsham 2.741349 1.5724689 298 1.743341 0.0823

visit9:Treatmentsham 3.294907 1.5148754 298 2.175035 0.0304

visit10:Treatmentsham 2.283569 1.5414275 298 1.481464 0.1395

visit11:Treatmentsham 3.476974 1.5558007 298 2.234845 0.0262

visit1:Treatmentactive:SiteMindLight -0.102564 1.4492025 298 -0.070773 0.9436

visit2:Treatmentactive:SiteMindLight 0.803796 1.4745260 298 0.545122 0.5861

visit3:Treatmentactive:SiteMindLight -1.144748 1.4540372 298 -0.787290 0.4317

visit4:Treatmentactive:SiteMindLight -0.099381 1.4559723 298 -0.068258 0.9456

visit5:Treatmentactive:SiteMindLight 0.120844 1.5250579 298 0.079239 0.9369

visit6:Treatmentactive:SiteMindLight -0.026454 1.4602893 298 -0.018116 0.9856

visit7:Treatmentactive:SiteMindLight 0.844892 1.4671011 298 0.575892 0.5651

visit8:Treatmentactive:SiteMindLight 0.232355 1.5233201 298 0.152532 0.8789

visit9:Treatmentactive:SiteMindLight 0.332848 1.4705596 298 0.226341 0.8211

visit10:Treatmentactive:SiteMindLight 0.245912 1.5141472 298 0.162409 0.8711

visit11:Treatmentactive:SiteMindLight 0.365631 1.5303119 298 0.238926 0.8113

visit1:Treatmentsham:SiteMindLight -0.740741 1.1548889 298 -0.641396 0.5218

visit2:Treatmentsham:SiteMindLight 0.479260 1.1665412 298 0.410839 0.6815

visit3:Treatmentsham:SiteMindLight 0.379591 1.1758317 298 0.322827 0.7471

visit4:Treatmentsham:SiteMindLight 1.086305 1.1792132 298 0.921212 0.3577

visit5:Treatmentsham:SiteMindLight 1.159719 1.1993518 298 0.966955 0.3344

visit6:Treatmentsham:SiteMindLight 0.787071 1.1903589 298 0.661204 0.5090

visit7:Treatmentsham:SiteMindLight 0.488778 1.1931897 298 0.409640 0.6824

visit8:Treatmentsham:SiteMindLight 0.334562 1.2105631 298 0.276369 0.7825

visit9:Treatmentsham:SiteMindLight 0.644683 1.2123822 298 0.531749 0.5953

visit10:Treatmentsham:SiteMindLight 0.720834 1.2063399 298 0.597538 0.5506

visit11:Treatmentsham:SiteMindLight 0.225152 1.2124475 298 0.185700 0.8528

**3. Use - TimeLine FollowBack minus Baseline**

Variables

+ Total.Opioid.Lookback...Base.2 – total opioid use minus baseline (numeric)

+ baseline.opioid.use – assessed opioid use prior to first treatment (numeric)

+ suboxone y or n for medication management with buprenorphine or suboxone (factor)

autoRegressive.tlfb5.weighted <- lme(Total.Opioid.Lookback...Base.2 ~ Treatment * visit * Site + suboxone + baseline.opioid.use, random = ~1 | Pt_ID, data = f1, correlation=corAR1(0, form = ~visitid_distinct|Pt_ID), weights = varIdent(form = ~1 | Treatment), method = "ML", na.action = na.exclude)

anova.lme(autoRegressive.tlfb5.weighted, type="marginal", adjustSigma=FALSE)

numDF denDF F-value p-value

(Intercept) 1 292 2.296764 0.1307

Treatment 1 33 2.124778 0.1544

visit 10 292 10.039327 <.0001

Site 1 33 3.243010 0.0809

suboxone 1 33 5.346248 0.0271

baseline.opioid.use 1 33 21.457671 0.0001

Treatment:visit 10 292 7.145438 <.0001

Treatment:Site 1 33 2.918775 0.0969

visit:Site 10 292 7.383006 <.0001

Treatment:visit:Site 10 292 5.279024 <.0001

Linear mixed-effects model fit by maximum likelihood

Data: f1

AIC BIC logLik

1065.643 1261.453 -482.8216

Random effects:

Formula: ~1 | Pt_ID

(Intercept) Residual

StdDev: 0.7868703 0.9449877

Correlation Structure: ARMA(1,0)

Formula: ~visitid_distinct | Pt_ID

Parameter estimate(s):

Phi1

0.2488356

Variance function:

Structure: Different standard deviations per stratum

Formula: ~1 | Treatment

Parameter estimates:

active sham

1.0000000 0.7721444

Fixed effects: Total.Opioid.Lookback...Base.2 ~ Treatment * visit * Site + suboxone + baseline.opioid.use

Value Std.Error DF t-value p-value

(Intercept) 0.865568 0.6102225 292 1.418446 0.1571

Treatmentsham -0.941261 0.6899198 33 -1.364305 0.1817

visit2 -4.082423 0.5352538 292 -7.627078 0.0000

visit3 -3.333333 0.5645871 292 -5.904020 0.0000

visit4 -3.166667 0.5784143 292 -5.474738 0.0000

visit5 -2.825508 0.6478289 292 -4.361503 0.0000

visit6 -4.084233 0.6112068 292 -6.682244 0.0000

visit7 -4.166667 0.5828533 292 -7.148740 0.0000

visit8 -3.876436 0.6496113 292 -5.967317 0.0000

visit9 -3.847495 0.6155003 292 -6.251004 0.0000

visit10 -3.826607 0.6567535 292 -5.826550 0.0000

visit11 -3.821409 0.6608634 292 -5.782450 0.0000

SiteMindLight -1.169480 0.6938473 33 -1.685501 0.1013

suboxoney 0.727735 0.3362747 33 2.164110 0.0378

baseline.opioid.use -0.415555 0.0958478 33 -4.335568 0.0001

Treatmentsham:visit2 3.978382 0.6401450 292 6.214813 0.0000

Treatmentsham:visit3 3.218120 0.6925174 292 4.646988 0.0000

Treatmentsham:visit4 3.032180 0.7013415 292 4.323401 0.0000

Treatmentsham:visit5 2.776936 0.7768274 292 3.574714 0.0004

Treatmentsham:visit6 4.282346 0.7483354 292 5.722496 0.0000

Treatmentsham:visit7 4.032936 0.7256983 292 5.557317 0.0000

Treatmentsham:visit8 3.719142 0.7706762 292 4.825817 0.0000

Treatmentsham:visit9 4.021542 0.7511501 292 5.353846 0.0000

Treatmentsham:visit10 3.807544 0.7854399 292 4.847657 0.0000

Treatmentsham:visit11 4.529023 0.7801397 292 5.805400 0.0000

Treatmentsham:SiteMindLight 1.405682 0.8790872 33 1.599024 0.1193

visit2:SiteMindLight 4.159346 0.6358474 292 6.541422 0.0000

visit3:SiteMindLight 3.276170 0.6869403 292 4.769221 0.0000

visit4:SiteMindLight 3.493650 0.7043722 292 4.959949 0.0000

visit5:SiteMindLight 2.984671 0.7689220 292 3.881631 0.0001

visit6:SiteMindLight 4.074429 0.7338333 292 5.552254 0.0000

visit7:SiteMindLight 4.192554 0.7154977 292 5.859633 0.0000

visit8:SiteMindLight 3.811051 0.7715458 292 4.939501 0.0000

visit9:SiteMindLight 4.098626 0.7437088 292 5.511063 0.0000

visit10:SiteMindLight 4.488984 0.7837952 292 5.727241 0.0000

visit11:SiteMindLight 3.877304 0.7877209 292 4.922180 0.0000

Treatmentsham:visit2:SiteMindLight -4.418941 0.7814073 292 -5.655106 0.0000

Treatmentsham:visit3:SiteMindLight -3.101967 0.8610845 292 -3.602395 0.0004

Treatmentsham:visit4:SiteMindLight -3.255099 0.8759233 292 -3.716192 0.0002

Treatmentsham:visit5:SiteMindLight -2.958827 0.9406932 292 -3.145368 0.0018

Treatmentsham:visit6:SiteMindLight -3.936179 0.9138918 292 -4.307051 0.0000

Treatmentsham:visit7:SiteMindLight -3.604278 0.8996066 292 -4.006504 0.0001

Treatmentsham:visit8:SiteMindLight -3.707716 0.9394895 292 -3.946522 0.0001

Treatmentsham:visit9:SiteMindLight -4.434196 0.9240603 292 -4.798600 0.0000

Treatmentsham:visit10:SiteMindLight -4.449233 0.9569860 292 -4.649215 0.0000

Treatmentsham:visit11:SiteMindLight -4.711881 0.9534090 292 -4.942140 0.0000

**4. Hamilton Depression Rating Scale (HDRS)**

Variables

+HDRS.Base – HDRS minus baseline (numeric)

+HDRS.baseline – HDRS score prior to first treatment (numeric)

hdrs6 <- lme(HDRS.Base ~ visit + Treatment:visit + Treatment:visit:suboxone + HDRS.baseline, random = ~1 | Pt_ID, correlation=corARMA(form = ~visitid_distinct|Pt_ID,p=2), data = f1, method = "ML", na.action = na.exclude)

anova.lme(movingAverage2.hdrs6, type="marginal", adjustSigma=FALSE)

numDF denDF F-value p-value

(Intercept) 1 292 3.526034 0.0614

visit 10 292 1.483471 0.1447

HDRS.baseline 1 37 30.628460 <.0001

visit:Treatment 11 292 1.470916 0.1416

visit:Treatment:suboxone 22 292 1.585291 0.0485

Linear mixed-effects model fit by maximum likelihood

Data: f1

AIC BIC logLik

2296.502 2488.79 -1099.251

Random effects:

Formula: ~1 | Pt_ID

(Intercept) Residual

StdDev: 2.506091 4.665533

Correlation Structure: ARMA(2,0)

Formula: ~visitid_distinct | Pt_ID

Parameter estimate(s):

Phi1 Phi2

0.2250331 0.2381545

Fixed effects: HDRS.Base ~ visit + Treatment:visit + Treatment:visit:suboxone + HDRS.baseline

Value Std.Error DF t-value p-value

(Intercept) 3.187528 1.8098749 292 1.761187 0.0793

visit2 -2.538100 1.8328090 292 -1.384814 0.1672

visit3 -3.727273 1.7687540 292 -2.107287 0.0359

visit4 -4.499785 2.0200440 292 -2.227568 0.0267

visit5 -4.891618 2.1773929 292 -2.246548 0.0254

visit6 -4.879992 2.1198333 292 -2.302064 0.0220

visit7 -5.550291 2.1416485 292 -2.591597 0.0100

visit8 -5.132357 2.2761667 292 -2.254825 0.0249

visit9 -1.641459 2.2286564 292 -0.736524 0.4620

visit10 -5.120250 2.3091584 292 -2.217366 0.0274

visit11 -3.434600 2.3187418 292 -1.481234 0.1396

HDRS.baseline -0.294645 0.0567643 37 -5.190686 0.0000

visit1:Treatmentsham 0.242987 2.2755583 292 0.106781 0.9150

visit2:Treatmentsham 1.686389 2.3417354 292 0.720145 0.4720

visit3:Treatmentsham 1.266234 2.2994940 292 0.550658 0.5823

visit4:Treatmentsham 3.219686 2.3440318 292 1.373567 0.1706

visit5:Treatmentsham 5.015504 2.4746549 292 2.026749 0.0436

visit6:Treatmentsham 5.603389 2.3827715 292 2.351627 0.0194

visit7:Treatmentsham 5.913832 2.3568057 292 2.509257 0.0126

visit8:Treatmentsham 6.752883 2.5215288 292 2.678091 0.0078

visit9:Treatmentsham 1.746271 2.4413107 292 0.715300 0.4750

visit10:Treatmentsham 4.873990 2.5161971 292 1.937046 0.0537

visit11:Treatmentsham 1.295248 2.5254988 292 0.512868 0.6084

visit1:Treatmentactive:suboxoney 0.421879 2.6250050 292 0.160715 0.8724

visit2:Treatmentactive:suboxoney 3.334979 2.6605467 292 1.253494 0.2110

visit3:Treatmentactive:suboxoney 2.774151 2.6250050 292 1.056818 0.2915

visit4:Treatmentactive:suboxoney 4.921663 2.6647247 292 1.846969 0.0658

visit5:Treatmentactive:suboxoney 3.441009 2.8201720 292 1.220141 0.2234

visit6:Treatmentactive:suboxoney 3.176870 2.6729613 292 1.188521 0.2356

visit7:Treatmentactive:suboxoney 3.762973 2.7391492 292 1.373774 0.1706

visit8:Treatmentactive:suboxoney 1.054235 2.7704234 292 0.380532 0.7038

visit9:Treatmentactive:suboxoney 1.188338 2.7247239 292 0.436131 0.6631

visit10:Treatmentactive:suboxoney 2.792129 2.7863613 292 1.002070 0.3171

visit11:Treatmentactive:suboxoney 1.617591 2.8625260 292 0.565092 0.5724

visit1:Treatmentsham:suboxoney 0.056123 2.7552759 292 0.020369 0.9838

visit2:Treatmentsham:suboxoney -2.164526 2.9260820 292 -0.739735 0.4601

visit3:Treatmentsham:suboxoney -5.601038 3.0865885 292 -1.814637 0.0706

visit4:Treatmentsham:suboxoney -4.832806 3.1023919 292 -1.557768 0.1204

visit5:Treatmentsham:suboxoney -7.871143 3.1270385 292 -2.517124 0.0124

visit6:Treatmentsham:suboxoney -5.408750 3.1369432 292 -1.724210 0.0857

visit7:Treatmentsham:suboxoney -10.156697 3.1176418 292 -3.257814 0.0013

visit8:Treatmentsham:suboxoney -12.526269 3.0004550 292 -4.174790 0.0000

visit9:Treatmentsham:suboxoney -8.421198 3.1353823 292 -2.685860 0.0076

visit10:Treatmentsham:suboxoney -7.391055 3.1526428 292 -2.344400 0.0197

visit11:Treatmentsham:suboxoney -5.559646 2.9850846 292 -1.862475 0.0635

***In participants not receiving suboxone***

anova.lme(hdrs6.no.sub , type="marginal", adjustSigma=FALSE)

numDF denDF F-value p-value

(Intercept) 1 194 1.997333 0.1592

visit 10 194 1.732356 0.0759

HDRS.baseline 1 23 10.412930 0.0037

visit:Treatment 11 194 2.060828 0.0249

**5. Hamilton Anxiety Rating Scale**

Variables

+HARS.Base – HARS – baseline (numeric)

+Hars.baseline – Baseline HARS scores before initial treatment (numeric)

movingAverage2.hars6 <- lme(HARS.Base ~ visit + visit:Treatment + HARS.baseline, random = ~1 | Pt_ID, correlation=corARMA(form = ~visitid_distinct|Pt_ID,p=2), data = f1, method = "ML", na.action = na.exclude)

anova.lme(movingAverage2.hars6, type="marginal", adjustSigma=FALSE)

numDF denDF F-value p-value

(Intercept) 1 314 5.395811 0.0208

visit 10 314 2.355254 0.0108

HARS.baseline 1 314 25.932711 <.0001

visit:Treatment 11 314 1.689394 0.0746

summary(movingAverage2.hars6)

Linear mixed-effects model fit by maximum likelihood

Data: f1

AIC BIC logLik

2237.179 2343.206 -1091.589

Random effects:

Formula: ~1 | Pt_ID

(Intercept) Residual

StdDev: 1.519415 4.90642

Correlation Structure: ARMA(2,0)

Formula: ~visitid_distinct | Pt_ID

Parameter estimate(s):

Phi1 Phi2

0.3225125 0.2501384

Fixed effects: HARS.Base ~ visit + visit:Treatment + HARS.baseline

Value Std.Error DF t-value p-value

(Intercept) 3.075803 1.3667038 314 2.250526 0.0251

visit2 -2.433802 1.2612743 314 -1.929638 0.0546

visit3 -3.608980 1.3035137 314 -2.768655 0.0060

visit4 -3.024037 1.4591264 314 -2.072498 0.0390

visit5 -4.925003 1.5770036 314 -3.123013 0.0020

visit6 -6.081696 1.5702794 314 -3.873003 0.0001

visit7 -5.216591 1.6173149 314 -3.225464 0.0014

visit8 -6.719609 1.6820616 314 -3.994865 0.0001

visit9 -4.607346 1.6585977 314 -2.777857 0.0058

visit10 -4.497301 1.7148687 314 -2.622534 0.0092

visit11 -5.295935 1.7272518 314 -3.066105 0.0024

HARS.baseline -0.232830 0.0471909 314 -4.933779 0.0000

visit1:Treatmentsham -0.537959 1.7018777 314 -0.316097 0.7521

visit2:Treatmentsham 1.818066 1.7343820 314 1.048250 0.2953

visit3:Treatmentsham 0.596282 1.7639319 314 0.338041 0.7356

visit4:Treatmentsham 1.127370 1.7520520 314 0.643457 0.5204

visit5:Treatmentsham 4.103610 1.8246060 314 2.249039 0.0252

visit6:Treatmentsham 6.040056 1.7759466 314 3.401035 0.0008

visit7:Treatmentsham 3.599385 1.7930115 314 2.007452 0.0456

visit8:Treatmentsham 4.097460 1.8517196 314 2.212787 0.0276

visit9:Treatmentsham 3.526735 1.8176866 314 1.940233 0.0532

visit10:Treatmentsham 3.133059 1.8681372 314 1.677103 0.0945

visit11:Treatmentsham 1.489520 1.8611650 314 0.800316 0.4241

**6. PANAS Positive**

Variables

**+** pre.PANAS.Pos..base – Initial positive affect PANAS ratings each visit prior to Rx minus baseline

**+** Baseline.PANAS.Pos1 – Baseline positive affect PANAS prior to any Rx

autoRegressive.pnas.p6 <- lme(pre.PANAS.Pos..base ~ Treatment + Ethnicity + Baseline.PANAS.Pos1, random = ~1 | Pt_ID, correlation=corAR1(0, form = ~visitid_distinct|Pt_ID), data = f1, method = "ML", na.action = na.exclude)

anova.lme(autoRegressive.pnas.p6, type="marginal", adjustSigma=FALSE)

numDF denDF F-value p-value

(Intercept) 1 333 14.023650 0.0002

Treatment 1 35 2.108679 0.1554

Ethnicity 1 35 6.715400 0.0138

Baseline.PANAS.Pos1 1 35 8.757187 0.0055

Linear mixed-effects model fit by maximum likelihood

Data: f1

AIC BIC logLik

2237.179 2343.206 -1091.589

Random effects:

Formula: ~1 | Pt_ID

(Intercept) Residual

StdDev: 1.519415 4.90642

Correlation Structure: ARMA(2,0)

Formula: ~visitid_distinct | Pt_ID

Parameter estimate(s):

Phi1 Phi2

0.3225125 0.2501384

Fixed effects: HARS.Base ~ visit + visit:Treatment + HARS.baseline

Value Std.Error DF t-value p-value

(Intercept) 3.075803 1.3667038 314 2.250526 0.0251

visit2 -2.433802 1.2612743 314 -1.929638 0.0546

visit3 -3.608980 1.3035137 314 -2.768655 0.0060

visit4 -3.024037 1.4591264 314 -2.072498 0.0390

visit5 -4.925003 1.5770036 314 -3.123013 0.0020

visit6 -6.081696 1.5702794 314 -3.873003 0.0001

visit7 -5.216591 1.6173149 314 -3.225464 0.0014

visit8 -6.719609 1.6820616 314 -3.994865 0.0001

visit9 -4.607346 1.6585977 314 -2.777857 0.0058

visit10 -4.497301 1.7148687 314 -2.622534 0.0092

visit11 -5.295935 1.7272518 314 -3.066105 0.0024

HARS.baseline -0.232830 0.0471909 314 -4.933779 0.0000

visit1:Treatmentsham -0.537959 1.7018777 314 -0.316097 0.7521

visit2:Treatmentsham 1.818066 1.7343820 314 1.048250 0.2953

visit3:Treatmentsham 0.596282 1.7639319 314 0.338041 0.7356

visit4:Treatmentsham 1.127370 1.7520520 314 0.643457 0.5204

visit5:Treatmentsham 4.103610 1.8246060 314 2.249039 0.0252

visit6:Treatmentsham 6.040056 1.7759466 314 3.401035 0.0008

visit7:Treatmentsham 3.599385 1.7930115 314 2.007452 0.0456

visit8:Treatmentsham 4.097460 1.8517196 314 2.212787 0.0276

visit9:Treatmentsham 3.526735 1.8176866 314 1.940233 0.0532

visit10:Treatmentsham 3.133059 1.8681372 314 1.677103 0.0945

visit11:Treatmentsham 1.489520 1.8611650 314 0.800316 0.4241

**7. PNAS Negative**

Variables

+ pre.PANAS.Negative..Base - Initial negative affect PANAS ratings each visit prior to Rx minus baseline

+ Baseline.pre.PANAS.Neg - Baseline positive affect PANAS prior to any Rx

movingAverage2.pnas.n8<- lme(pre.PANAS.Negative..Base ~ Site + Ethnicity + Employment + Baseline.pre.PANAS.Neg, random = ~1 | Pt_ID, correlation=corARMA(form = ~visitid_distinct|Pt_ID,p=2), data = f1, method = "ML", na.action = na.exclude)

anova.lme(movingAverage2.pnas.n8, type="marginal", adjustSigma=FALSE)

numDF denDF F-value p-value

(Intercept) 1 332 6.13203 0.0138

Site 1 35 2.80345 0.1030

Ethnicity 1 35 6.17183 0.0179

Employment 1 35 2.67677 0.1108

Baseline.pre.PANAS.Neg 1 332 44.10127 <.0001

summary(movingAverage2.pnas.n8)

Linear mixed-effects model fit by maximum likelihood

Data: f1

AIC BIC logLik

1672.027 1707.297 -827.0135

Random effects:

Formula: ~1 | Pt_ID

(Intercept) Residual

StdDev: 1.428414 2.283407

Correlation Structure: ARMA(2,0)

Formula: ~visitid_distinct | Pt_ID

Parameter estimate(s):

Phi1 Phi2

0.2453385 0.2420854

Fixed effects: pre.PANAS.Negative..Base ~ Site + Ethnicity + Employment + Baseline.pre.PANAS.Neg

Value Std.Error DF t-value p-value

(Intercept) 2.4236886 0.9854010 332 2.459596 0.0144

SiteMindLight 1.0294637 0.6190181 35 1.663059 0.1052

Ethnicityw 1.5420225 0.6249168 35 2.467564 0.0186

Employmenty -1.0807605 0.6650627 35 -1.625050 0.1131

Baseline.pre.PANAS.Neg -0.5623864 0.0852605 332 -6.596098 0.0000

**8. Wellness**

Variables

**+** pre.Wellbeing..Base - Initial wellness ratings each visit prior to Rx minus baseline

+ pre.Wellbeing.Base - Baseline wellness ratings prior to any Rx

movingAverage2.well8 <- lme(pre.Wellbeing..Base ~ Ethnicity + Employment + pre.Wellbeing.Base, random = ~1 | Pt_ID, data = f1, method = "ML", na.action = na.exclude)

anova.lme(movingAverage2.well8, type="marginal", adjustSigma=FALSE)

numDF denDF F-value p-value

(Intercept) 1 336 35.76518 <.0001

Ethnicity 1 35 6.17595 0.0179

Employment 1 35 3.99643 0.0534

pre.Wellbeing.Base 1 35 39.10963 <.0001

summary(movingAverage2.well8)

Linear mixed-effects model fit by maximum likelihood

Data: f1

AIC BIC logLik

1372.899 1404.315 -678.4496

Random effects:

Formula: ~1 | Pt_ID

(Intercept) Residual

StdDev: 0.6612934 1.584207

Correlation Structure: ARMA(2,0)

Formula: ~visitid_distinct | Pt_ID

Parameter estimate(s):

Phi1 Phi2

0.3112093 0.2239246

Fixed effects: pre.Wellbeing..Base ~ Ethnicity + Employment + pre.Wellbeing.Base

Value Std.Error DF t-value p-value

(Intercept) 3.631979 0.6105788 336 5.948419 0.0000

Ethnicityw -0.887351 0.3589816 35 -2.471856 0.0185

Employmenty 0.752842 0.3786141 35 1.988416 0.0546

pre.Wellbeing.Base -0.488436 0.0785226 35 -6.220327 0.0000

**9. Distress**

Variables

**+** pre.Distress..BASE - Initial distress ratings each visit prior to Rx minus baseline

+ pre.Wellbeing.Base - Baseline distress ratings prior to any Rx

movingAverage2.distress6, <- lme(pre.Distress..BASE ~ visit + visit:Treatment + Ethnicity + Employment + pre.Distress.Base, random = ~1 | Pt_ID, correlation=corARMA(form = ~visitid_distinct|Pt_ID,p=2), data = f1, method = "ML", na.action = na.exclude)

anova.lme(movingAverage2.distress6, type="marginal", adjustSigma=FALSE)

numDF denDF F-value p-value

(Intercept) 1 332 2.63755 0.1053

visit 10 332 4.31467 <.0001

Ethnicity 1 35 7.25615 0.0108

Employment 1 35 3.05294 0.0894

pre.Distress.Base 1 35 53.64537 <.0001

visit:Treatment 11 332 2.61241 0.0033

summary(movingAverage2.distress6)

Linear mixed-effects model fit by maximum likelihood

Data: f1

AIC BIC logLik

1544.07 1659.236 -743.0348

Random effects:

Formula: ~1 | Pt_ID

(Intercept) Residual

StdDev: 0.3122648 1.676659

Correlation Structure: ARMA(2,0)

Formula: ~visitid_distinct | Pt_ID

Parameter estimate(s):

Phi1 Phi2

0.1754151 0.2435229

Fixed effects: pre.Distress..BASE ~ visit + visit:Treatment + Ethnicity + Employment + pre.Distress.Base

Value Std.Error DF t-value p-value

(Intercept) -0.7207662 0.4586737 332 -1.571414 0.1170

visit2 -0.9281256 0.5004408 332 -1.854616 0.0645

visit3 0.6315789 0.4756519 332 1.327818 0.1852

visit4 0.5789474 0.5314760 332 1.089320 0.2768

visit5 1.4134917 0.5591263 332 2.528036 0.0119

visit6 1.5789474 0.5504699 332 2.868363 0.0044

visit7 1.4210526 0.5540933 332 2.564645 0.0108

visit8 1.7335617 0.5799525 332 2.989144 0.0030

visit9 1.2255473 0.5668049 332 2.162203 0.0313

visit10 0.3370736 0.5763662 332 0.584825 0.5591

visit11 1.8570113 0.5859544 332 3.169208 0.0017

Ethnicityw -0.7484218 0.2871463 35 -2.606413 0.0134

Employmenty 0.5228110 0.3092398 35 1.690633 0.0998

pre.Distress.Base 0.3967191 0.0559792 35 7.086897 0.0000

visit1:Treatmentsham -0.1897904 0.5678478 332 -0.334228 0.7384

visit2:Treatmentsham 1.4024691 0.5814806 332 2.411893 0.0164

visit3:Treatmentsham 0.3753549 0.5813454 332 0.645666 0.5189

visit4:Treatmentsham -0.3710436 0.5755611 332 -0.644664 0.5196

visit5:Treatmentsham -1.5939035 0.6085526 332 -2.619172 0.0092

visit6:Treatmentsham -1.4464085 0.5893852 332 -2.454097 0.0146

visit7:Treatmentsham -0.2839102 0.5892070 332 -0.481851 0.6302

visit8:Treatmentsham -0.4962873 0.6098125 332 -0.813836 0.4163

visit9:Treatmentsham -0.2184756 0.5962995 332 -0.366386 0.7143

visit10:Treatmentsham 1.1088823 0.6051928 332 1.832279 0.0678

visit11:Treatmentsham 0.0052318 0.6064689 332 0.008627 0.9931
